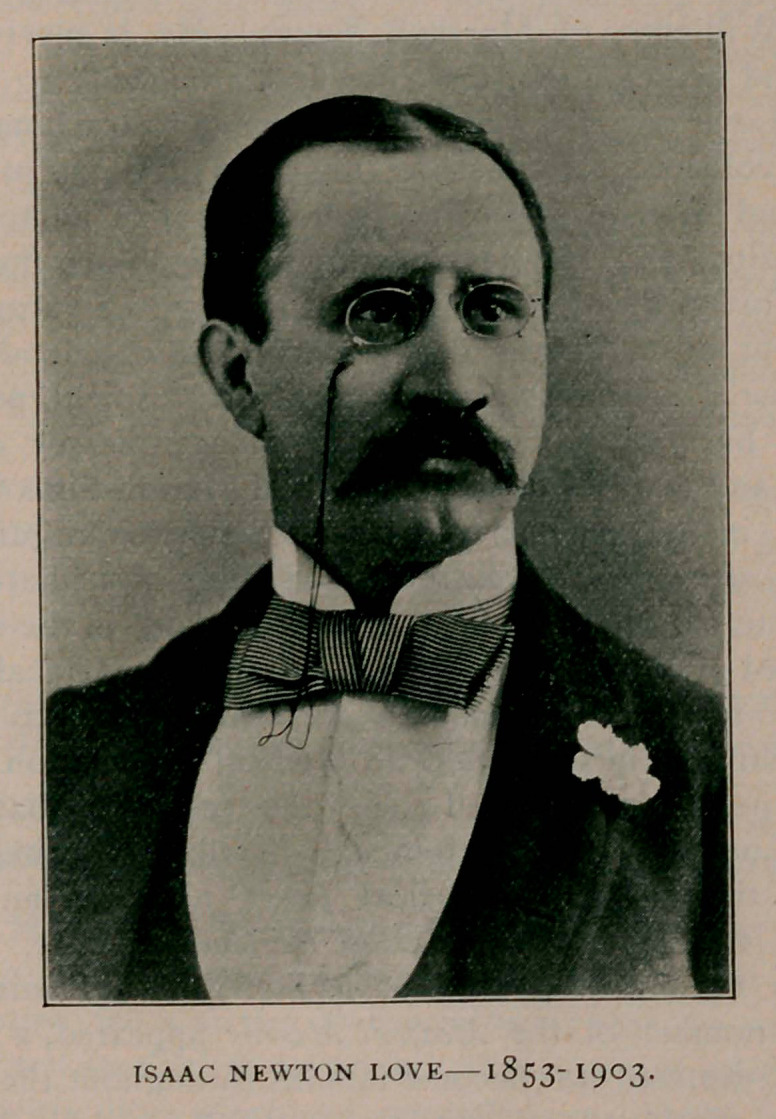# Dr. Isaac Newton Love

**Published:** 1903-07

**Authors:** 


### OBITUARY.

Dr. Isaac Newton Love, of New York, died June 18, 1903, from apoplexy on the Cunard steamer "Aurania," while she was at quarantine, just before coming up the bay to her pier. Dr. Love was in the saloon of the steamship, speaking of the pleasant trip, when he suddenly fell to the floor. He was immediately carried to his stateroom, where he was attended by the ship's surgeon. He died within ten minutes after he was stricken.

Dr. Love sailed for France about a month ago with a patient, Mrs. George Law. Leaving her there, he started for home, returning by way of England. The voyage across was an unusually pleasant one. Dr. Love, who was a good entertainer, was reported to have been the life of the ship, and helped greatly to make the time pass pleasantly. A committee, of which he was chairman, was selected to prepare and read to

Captain Potter a set of resolutions expressing the pleasure which had been experienced. Dr. Love was speaking informally, after reading the resolutions, when he was stricken. His body was taken to his home, the Nevada apartments, Broadway and Sixty-ninth Street, and the funeral was held from Saint Paul's Methodist Episcopal Church, at Eighty-sixth Street and West End Avenue, Monday afternoon, June 22.

ISAAC NEWTON LOVE—1853-1903.

Dr. Love was born at Barry, Pike County, Ill., September 13, 1853, and was therefore in the fiftieth year of his age at the time of his death. His parents were Isaac N. and Nancy Love. His father having died when he was two years old, and his mother ten years later, he was sent to Saint Louis to live with Dr. John T. Hodgen, a relative, and then the leading surgeon of that city. He began the study of medicine early in life, and received his doctorate degree in 1872 from Saint Louis Medical College. Soon after his graduation, through competitive examination open to all the graduates of Saint Louis medical schools, he received the appointment of assistant resident physician at the Saint Louis city hospital. Still later, he was appointed city physician in the administration of Mayor James H. Britton, in which office he served one year, after which he engaged in private practice, which continued to occupy his attention until his death.

In 1878, Dr. Love married Florence N., daughter of Judge John F. Williams, of Marshall, Texas. He is survived by his widow and two children, Delphine and Hodgen.

Dr. Love received many appointments to teaching chairs in medical colleges. At first he taught physiology in his *alma mater*, and afterward became demonstrator of anatomy in the same institution. He developed a strong liking for children early in his professional career and, owing to a vacancy in the department of children's diseases in the college, he determined to become a specialist in pediatrics. In this field, perhaps, he achieved his greatest fame. He became professor of clinical medicine and diseases of children in the Marion-Sims College of Medicine in 1890, when that institution was organised. In 1887, he served as secretary of the section on pediatrics in the Ninth International Medical Congress, and in the same year was elected president of the Mississippi Valley Medical Association. In 1889, he was chosen president of the section on diseases of children in the American Medical Association, and also in the same year was elected one of the trustees of that body.

Dr. Love had been a frequent contributor during all these years to the medical periodical press, and was an associate editor of the *Saint Louis Medical Review*. Finally, he determined to establish a journal of his own, and in January, 1890, the first number of the *Medical Mirror* appeared, a magazine that was destined to become famous throughout the land and which he continued to edit and publish until his death. In 1893, he was elected vice-president of the American Medical Association and also served as an officer of the first Pan-American Medical Congress, held at Washington, in September, 1893. About three years ago, in order to broaden his field of labor, he removed to New York City, where he continued in the active practice of his profession until his sudden demise.

Dr. Love was a well-equipped physician both for teaching and practice. His original methods in the lecture room attracted attention early in his professorial career, and his popularity was promptly established with both colleagues and pupils. As a clinician his talents were developed in a remarkable degree, his diagnostic acumen being not second to his therapeutic skill, and his manner in the sick room gained for him the deep attachment of his patients. He was, moreover, a genial companion, a staunch friend, and a broadly cultured man. A raconteur of the first order, he was capable of entertaining on any occasion when wit, story or eloquence, one or all, were called for. His versatility was such that he could easily adapt himself to the grave or gay, though his sunny nature would not brook gravity for very long. He could discuss science, art or literature, in the most entertaining, sober manner; and in another instant provoke his auditors to exceeding mirth by bright saying, witty anecdote, or clever speech; indeed, singularly enough and altogether fitting, it was while exercising these gifts that the messenger came and summoned him to the hereafter. Could he have chosen he would have had it thus.

## Figures and Tables

**Figure f1:**